# Reproductive aging and MCM8/9

**DOI:** 10.18632/oncotarget.4589

**Published:** 2015-06-22

**Authors:** Svetlana A. Yatsenko, Aleksandar Rajkovic

**Affiliations:** Magee-Women's Research Institute, University of Pittsburgh, Pittsburgh, Pennsylvania, USA

**Keywords:** reproductive aging, MCM8, MCM9, chromosomal instability, ovarian dysgenesis

Inherited defects in DNA repair are rare genetic conditions characterized by hypersensitivity to endogenous and exogenous DNA damage. Double-strand break (DSB) is the most severe form of DNA damage which can induce gross chromosomal rearrangements such as translocations, deletions, duplications, and complex genomic aberrations, triggering permanent cell cycle arrest and, ultimately, the death or malignant transformation of the affected cells. Hereditary defects in cellular DNA repair processes cause syndromic recessive conditions such as Fanconi anemia, Ataxia-telangiectasia, Bloom syndrome, Werner, and Xeroderma pigmentosum. Mutations in DSB repair genes have pleiotropic effects and are associated with growth retardation, skin, bone marrow, immune, nervous systems, and endocrine dysfunction as well as predisposition to cancers. Remarkably, the majority of the patients susceptible to chromosome breakage and genome instability also present with early gonadal dysfunction.

**Figure 1:**
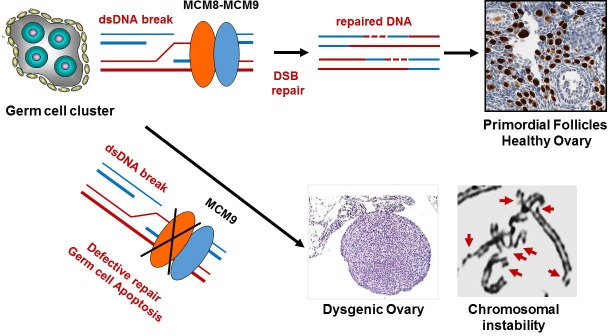
MCM8 and MCM9 presumed mechanism of action MCM8 and MCM9 physically interact to promote double stranded break repair. In the presence of functional MCM8 and MCM9, primordial follicles form, with oocytes stained brown using oocyte-specific anti-SOHLH1 antibodies. In the absence of MCM8 or MCM9, germ cell depletion occurs, leading to dysgenic ovary and chromosomal instability. Red arrows show representative chromosomal damage in stimulated T lymphocytes exposed to mitomycin C, from patients with homozygous *MCM8* or *MCM9* mutations.

We recently used whole exome sequencing to discover homozygous mutations in *MCM8* or *MCM9* genes in families whose members presented with absent or very small ovaries (ovarian dysgenesis) and chromosomal instability in somatic cells [1, 2]. Affected individuals in these families were diagnosed with hypergonadotropic (high follicle stimulating hormone levels) primary amenorrhea, hypothyroidism, growth retardation, and demonstrated a striking sensitivity to DNA cross-linking agents such as mitomycin C for both cultured fibroblasts and phytohaemagglutinin stimulated T-lymphocytes (Figure 1). The heterozygous mutation carriers appeared healthy and fertile, however they had an increased number of chromosome breakages in comparison to wild-type *MCM8* and *MCM9* individuals. None of the family members with homozygous mutations in *MCM8* or *MCM9* genes had cancer at the time of investigation, but all of them were premenopausal. Another group also reported additional families with homozygous mutations in *MCM8* and gonadal dysgenesis [3]. Mice deficient in *MCM8* or *MCM9* are also infertile due to gonadal dysgenesis, are susceptible to chromosomal breakage, and develop ovarian tumors of unknown significance [4, 5] We don't know whether humans with *MCM8* or *MCM9* mutations are susceptible to cancer, however, given animal models findings, it is prudent for such individuals to be followed in high risk clinic for regular cancer surveillance.

MCM8 and MCM9 proteins are members of the Mini-Chromosome Maintenance (MCM) protein family. This family also includes MCM2-MCM7 proteins that are known to be involved in DNA replication, but not in DSB repair. The current mouse and human findings show that MCM8 and MCM9 interact physically, and are integral to DSB repair. In gametogenesis, the excessive number of programmed DSBs is generated to attain accurate segregation of chromosomes. In general, only ~10% of them will result in homologous chromosomes crossovers, while the remaining DNA breaks must be repaired via homologous recombination (HR)-mediated mechanism to maintain germ-cells genomic integrity. The MCM8-MCM9 complex is rapidly recruited at the DNA-damaging sites, forming nuclear foci to resolve DSB and restore chromosomal integrity. The presence of DSB activates a cascade of DNA damage responses including checkpoint, cell cycle arrest, and DNA repair, while failure in any of these processes usually leads to cell apoptosis, depletion of germ cells and loss of ovarian reserve (Figure 1).

Mendelian disorders are relatively rare, but recent genome-wide association studies (GWAS) on large populations have strongly associated, a non-synonymous single nucleotide polymorphism (SNP, rs16991615, missense mutation in a coding exon 9) in the *MCM8* gene with the age of menopause in Caucasian, European and Hispanic women [6]. Moreover, meta-analysis of genome-wide association studies and age of menopause highlighted the preponderance of loci involved in DNA break repair [7]. *MCM9* is not one of the loci identified in GWAS studies, but this could be due to underpowered studies. Nonetheless, it is clear that *MCM8*, and by physical association, *MCM9*, may have far reaching effects on a significant segment of the population and their reproductive life span. These studies also argue that in a subset of individuals, accelerated aging is not caused by ovarian aging per se, rather by underlying susceptibility to chromosomal breakage. Ovarian dysfunction may therefore be a sensitive measure of overall propensity to age faster. Similar relationship between testicular dysfunction and overall aging may operate in a subset of men.
